# Differential roles of Smad2 and Smad3 in the regulation of TGF-β1-mediated growth inhibition and cell migration in pancreatic ductal adenocarcinoma cells: control by Rac1

**DOI:** 10.1186/1476-4598-10-67

**Published:** 2011-05-30

**Authors:** Hendrik Ungefroren, Stephanie Groth, Susanne Sebens, Hendrik Lehnert, Frank Gieseler, Fred Fändrich

**Affiliations:** 1 Clinic for Applied Cellular Medicine, University Hospital Schleswig-Holstein (UKSH) Campus Kiel, 24105 Kiel, Germany; 2 Institute of Experimental Medicine c/o Laboratory of Molecular Gastroenterology and Hepatology, Department of Internal Medicine I, University Hospital Schleswig-Holstein (UKSH) Campus Kiel, 24105 Kiel, Germany; 3 First Department of Medicine, University Hospital Schleswig-Holstein (UKSH), Campus Lübeck, 23538 Lübeck, Germany; 4 Current address: Department of Dermatology, UKSH, Campus Lübeck, 23538 Lübeck, Germany

## Abstract

**Background:**

Progression of pancreatic ductal adenocarcinoma (PDAC) is largely the result of genetic and/or epigenetic alterations in the transforming growth factor-beta (TGF-β)/Smad signalling pathway, eventually resulting in loss of TGF-β-mediated growth arrest and an increase in cellular migration, invasion, and metastasis. These cellular responses to TGF-β are mediated solely or partially through the canonical Smad signalling pathway which commences with activation of receptor-regulated Smads (R-Smads) Smad2 and Smad3 by the TGF-β type I receptor. However, little is known on the relative contribution of each R-Smad, the possible existence of functional antagonism, or the crosstalk with other signalling pathways in the control of TGF-β1-induced growth inhibition and cell migration. Using genetic and pharmacologic approaches we have inhibited in PDAC cells endogenous Smad2 and Smad3, as well as a potential regulator, the small GTPase Rac1, and have analysed the consequences for TGF-β1-mediated growth inhibition and cell migration (chemokinesis).

**Results:**

SiRNA-mediated silencing of Smad3 in the TGF-β responsive PDAC cell line PANC-1 reduced TGF-β1-induced growth inhibition but increased the migratory response, while silencing of Smad2 enhanced growth inhibition but decreased chemokinesis. Interestingly, siRNA-mediated silencing of the small GTPase Rac1, or ectopic expression of a dominant-negative Rac1 mutant largely mimicked the effect of Smad2 silencing on both TGF-β1-induced growth inhibition, via upregulation of the cdk inhibitor p21^WAF1^, and cell migration. Inhibition of Rac1 activation reduced both TGF-β1-induction of a Smad2-specific transcriptional reporter and Smad2 C-terminal phosphorylation in PDAC cells while Smad3-specific transcriptional activity and Smad3 C-terminal phosphorylation appeared increased. Disruption of autocrine TGF-β signalling in PANC-1 cells rendered cells less susceptible to the growth-suppressive effect of Rac1 inhibition, suggesting that the decrease in "basal" proliferation upon Rac1 inhibition was caused by potentiation of autocrine TGF-β growth inhibition.

**Conclusions:**

In malignant cells with a functional TGF-β signalling pathway Rac1 antagonizes the TGF-β1 growth inhibitory response and enhances cell migration by antagonistically regulating Smad2 *and *Smad3 activation. This study reveals that Rac1 is prooncogenic in that it can alter TGF-β signalling at the R-Smad level from a tumour-suppressive towards a tumour-promoting outcome. Hence, Rac1 might represent a viable target for therapeutic intervention to inhibit PDAC progression.

## Background

TGF-β and its signalling effectors regulate many aspects of tumour cell biology, such as growth arrest, and cell motility the latter of which is important for the metastatic dissemination of tumour cells from their primary location to lymph or blood vessels [1,2]. TGF-β's cellular activities are mediated by specific receptor complexes that are assembled upon ligand binding and comprise the TGF-β type II receptor (TβRII) and TGF-β type I receptor (TβRI/ALK5). The activated ligand-receptor complex typically activates the Smad signalling pathway. The canonical Smad signalling cascade is initiated by C-terminal phosphorylation of receptor-regulated Smad transcription factors (R-Smads) Smad2 and/or Smad3 by activated ALK5 [3]. This allows R-Smad binding to Smad4 and translocation of the complex to the nucleus where it can recruit transcriptional coactivators or corepressors to Smad binding elements (SBEs) in the promoters of TGF-β target genes [1,2]. The TGF-β signalling effectors are also key players of tumour cell behaviour and are often deregulated in cancer cells [2,4]. For instance, human pancreatic ductal adenocarcinoma (PDAC) is characterized besides the common K-Ras mutations (representing an early event in PDAC tumourigenesis) by both TGF-βoverexpression and mutational inactivation of the tumour suppressor Smad4/DPC4, the latter being a relatively late event. Recent studies in mice have shown that blockade of TGF-β/Smad signalling and activated Ras signalling cooperate to promote PDAC progression [5,6]. The crucial role of the Smad pathway in PDAC formation was also highlighted in orthotopic xenotransplantation experiments with TGF-β responsive PANC-1 cells, by which we demonstrated that Smad signalling through a kinase-active version of ALK5 suppressed primary tumour growth, but enhanced metastatic progression [7]. A recent study in breast cancer cells has revealed that TGF-β signalling was activated transiently and locally and caused a switch from cohesive movement to single cell motility and promoted haematogenous metastasis [8].

Smad2/3 and Smad4 are direct mediators of TGF-β signalling and there is now ample evidence to suggest that Smad2 and Smad3 have distinct and non-overlapping roles in TGF-β signalling and that these differ in epithelial cells and fibroblasts [reviewed in Ref. [9]]. However, relatively few studies on the roles of Smad2 and Smad3 in TGF-β signalling have been performed in human epithelial cells from which most cancers arise. Moreover, it remained a mystery why TGF-β can induce different functions, such as growth arrest and epithelial-to-mesenchymal transition (EMT), in the same cell lines, even though both play opposing roles in tumourigenesis [9]. The mechanisms for the selective activation of Smad2 *versus *Smad3 are largely unknown but can principally occur at the level of the TβRs, nuclear import and export, protein turnover, and/or at the transcriptional level. Alternatively, Smad2 *versus *Smad3 responses may be selected by post-translational modifications such as differential phosphorylation at the TβR complex [9]. It is possible that the availability of other factors such as co-repressors and co-activators determine which response is mediated by Smad3 and Smad2. Since strategies for therapeutic targeting of the TGF-β signalling pathway are being pursued, revealing the identity of factors that modulate the relative activation of Smad2 or Smad3 in the TGF-β response may provide target(s) for more effective strategies for cancer therapy.

Rac1 belongs to the Rho family of small GTPases and has been implicated in the organization of the actin cytoskeleton, the formation of lamellopodia and focal adhesions, and in endocytic vesicle trafficking and receptor endocytosis. Rac1 can also drive cell proliferation and protect cells from apoptosis through its ability to activate extracellular signal-regulated kinases (ERKs), phosphatidylinositol-3 kinase (PI3-K), and the transcription factor NFκB [reviewed in Ref. [10]]. Activated Rac1 acts synergistically with ligand-activated epidermal growth factor-receptor (EGF-R) to stimulate pancreatic tumour cell proliferation through cyclin D1 upregulation [11]. Rac1 has a critical role in cell migration, and in the invasive, and metastatic behavior of cancer cells [12-14]. Moreover, Rac1 function is required for oncogenic K-Ras tumourigenesis and proliferation [15]. Activation of Rac1 is accompanied by its rapid translocation from the cytosol to the cell membrane, where it exerts part of its effects as an essential subunit of the reactive oxygen species (ROS)-producing enzyme NAD(P)H oxidase [16]. In PDAC dysregulated expression of Rac1 was observed in the tumour cell compartment [17], along with high activity of Vav1, a guanine exchange factor (GEF), which exhibits a particularly strong guanine exchange activity for Rac1 [11]. Also TGF-β and Rac1 signalling exert antagonistic roles in tumour cell proliferation but share common nuclear targets such as cyclin D1 and p21^WAF1 ^[18,19]. Initial evidence for a role of Rac1 in TGF-β signalling came from transcriptional reporter gene assays with dominant negative (dn) and constitutively active (ca) mutants [20] and this was followed by the demonstration that Rac1 is involved in TGF-β-induced EMT [12]. We have shown earlier that Rac1 is rapidly activated following stimulation of PDAC cells with TGF-β1 and that dn inhibition of Rac1 activity blunted both TGF-β1-induced p38 MAPK activation and expression of the small leucine-rich proteoglycan biglycan [21].

As mentioned above, we demonstrated in orthotopic xenotransplantation experiments that Smad signalling through a kinase-active version of ALK5 suppressed primary tumour growth and enhanced metastatic progression [7]. However, the design of this study did not permit to test why Smad signalling exerted opposite effects on both responses and whether each response may be mediated predominantly or exclusively by only one of the two R-Smads. In this study we therefore asked whether growth inhibition and cell migration (as *in vitro *correlates of tumour growth and metastasis) are controlled differentially by Smad2 and Smad3 and whether Rac1 impacts on differential activation of both R-Smads by TGF-β1. For this purpose, we utilized the well characterized PDAC cell lines PANC-1 and COLO 357 which have retained a functional TGF-β/Smad pathway [4,22-24]. Using RNA interference to specifically deplete cells of the expression of the two R-Smads, we found that TGF-β1-induced growth inhibition was dependent on Smad3 (confirming earlier observations in PANC-1 cells [25]) while the migratory response to TGF-β1 was positively controlled by Smad2. We went on to show that Rac1 modulates TGF-β1-signalling in PDAC cells by suppressing and promoting, respectively, TGF-β1-induced activation of Smad3 and Smad2, eventually resulting in protection of PDAC cells from excessive growth inhibition by TGF-β1 and in enhanced cell migration (chemokinesis).

## Results

### Differential control of TGF-β1-induced growth inhibition, cell migration, and migration-associated gene expression by Smad3 and Smad2

Using RNA interference to selectively deplete Smad2 and Smad3, a previous study demonstrated that sensitivity to TGF-β growth-inhibitory signalling (as measured by cell counting and flow cytometry analyses) was dependent on the endogenous ratio of Smad2 and Smad3 in various cell lines including PANC-1 cells [25]. To confirm that this mechanism also operated in the PANC-1 cells used in our study and to verify functionality of Smad2 and Smad3 small interfering (si) RNAs, we transfected PANC-1 cells with these siRNAs and subsequently measured the growth response to a 24-h treatment with TGF-β1 using [^3^H]-thymidine incorporation (Additional file 1 Figure S1A). In keeping with the idea that in cells of epithelial origin TGF-β1 mediates its inhibitory effect on cell growth predominantly through Smad3 [9,25], silencing of Smad3 diminished the inhibitory growth response (Additional file 1 Figure S1A). Notably, however, in cells with silenced Smad2 the growth suppressive effect of TGF-β1 on DNA synthesis was strongly enhanced in a similar fashion (Additional file 1 Figure S1A). Specificity and selectivity of the siRNAs for the respective Smads was further confirmed in immunoblot analysis (Additional file 1 Figure S1A). As predicted, depletion of the total Smads also decreased the levels of the respective phospho-Smads (p-Smads) expressed constitutively (Additional file 1 Figure S1B) and after stimulation with exogenous TGF-β1 (not shown). Also of interest, the knockdown of Smad2 alone translated into higher expression of the cyclin-dependent kinase inhibitor p21^WAF1 ^as shown previously [25] (Additional file 1 Figure S1A), suggesting that Smad2 normally acts to suppress p21^WAF1^. These data show that TGF-β1-mediated antiproliferative signals in PANC-1 cells rely on a Smad3-, but not Smad2-, dependent pathway and that the degree of TGF-β1-induced growth inhibition can be enhanced by increasing the endogenous ratio of Smad3 to Smad2.

**Figure 1:**
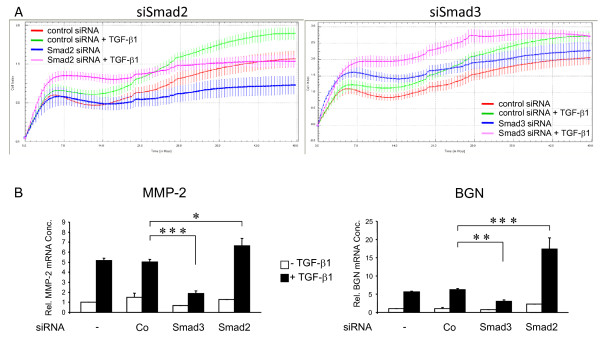
**Differential regulation of TGF-β1-induced cell migration and gene expression by Smad2 and Smad3 as revealed by siRNA-mediated knockdown experiments**. PANC-1 cells were transfected twice with siRNAs to Smads 2 and 3, or matched control siRNA, as described in the Method section and analysed for the cell's migratory response to TGF-β1 in a real-time cell migration (RTCA) assay (A) and for regulation of the migration-associated TGF-β1 target genes *MMP2 *and *BGN *(B). (A) The relative number of cells that have migrated over the indicated time period (abcissa) through the pores of the transwells and changed the impedance is given as cell index (ordinata). Data represent the mean ± s.d. of a representative experiment chosen from a series of three independent experiments. Data are significant because of the large number of independently measured time points (B) PANC-1 cells were transfected with transfection agent alone (-), control siRNA, or siRNAs against Smad3 or Smad2 (parallel samples from the experiment shown in A). Seventy-two hours after the first round of transfection cells were stimulated with TGF-β1 for 24 h and subjected to qPCR analysis for *MMP2 *and *BGN*. Data represent the mean ± standard deviation from triplicate wells after normalization with β-actin and TBP. One out of three experiments is shown. *, *p *< 0.05; **, *p *< 0.01; ***, *p *< 0.001.

The relative roles played by Smad2 and Smad3 in the control of basal and TGF-β1-induced cell motility (chemokinesis) in PDAC cells have not yet been uncovered. To do this, we transfected cells with siRNAs to Smads 2 and 3 as described above and analysed the cell's migratory response to TGF-β1 with a novel real-time-based cell migration assay (xCELLigence DP system). As seen in Figure 1A, PANC-1 cell migration showed an early (5-6 h) increase which reflected the high spontaneous migratory activity of these cells and which was largely independent of exogenously added TGF-β1 stimulation. This initial rise was followed by a more pronounced and long-lasting increase in migration which was sensitive to recombinant TGF-β1 and which peaked between 40 and 50 hrs (Figure 1A). PANC-1 cells (Figure 1A) and COLO 357 cells (Additional file 2 Figure S2B) transfected with Smad2 siRNA exhibited a basal and exogenous TGF-β1-triggered migratory activity that was clearly lower than that of mock-transfected cells (not shown) or cells that received a matched negative control siRNA. In contrast, under the same conditions the basal and TGF-β1-induced motility of Smad3 siRNA transfected cells exceeded that of the respective controls (Figure 1A). The finding that Smad3 inhibition failed to impair TGF-β1-induced chemokinesis was independently confirmed in COLO 357 cells with a pharmacologic Smad3 inhibitor (SIS3, [26]) that has been shown not to cross-inhibit Smad2 (Additional file 2 Figure S2A). These data show that TGF-β1-mediated promigratory signals in PDAC cells rely on a Smad2-, but not Smad3-, dependent pathway and that the intensity of TGF-β1-induced motility can be modulated by changing the endogenous ratio of Smad3 to Smad2. To test whether the differential and antagonistic regulation by Smad2 and Smad3 was also reflected at the level of individual genes functionally implicated in the control of TGF-β1-regulated cell migration/invasion, we analysed the response of the *MMP2 *and *BGN *genes (encoding matrix metalloprotease-2 and biglycan, respectively) in PANC-1 cells. Interestingly, knockdown of Smad3 suppressed, while knockdown of Smad2 potentiated the TGF-β1-induction of both *MMP2 *and *BGN *(Figure 1B).

### Specific depletion of Rac1 expression enhances growth inhibition induced by exogenous TGF-β1

**Figure 2:**
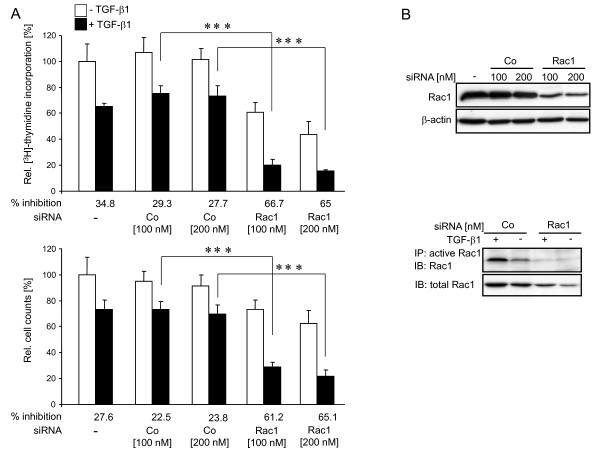
**Specific inhibition of Rac1 expression mimics the effect of Smad2 knockdown on basal proliferation and enhances growth inhibition induced by exogenous TGF-β1**. (A) PANC-1 cells were transfected with transfection agent alone (-), or with different amounts (as indicated) of either irrelevant control (Co) or Rac1-specific (Rac1) siRNAs for 3 h. After another 48 h in normal growth medium in the absence or presence of TGF-β1 cells were either counted (lower panel) or subjected to [^3^H]-thymidine incorporation assay (upper panel). The percentage of TGF-β1-induced growth inhibition relative to the respective untreated control is indicated below the bars. **Data represent the mean ± standard deviation from six wells (thymidine incorporation) or 3 wells (cell numbers) processed in parallel**. Shown is a representative experiment each from three independent experiments. Asterisks, *p *< 0.001. (B) A fraction of the cells from (A) were subjected to immunoblot (IB) analysis for Rac1 and β-actin as loading control (upper panel), or were stimulated with TGF-β1 for 15 min and subjected to immunoprecipitation (IP) of active Rac1 (lower panel). The active Rac1 was subsequently detected by immunoblotting (IB) with a Rac1 antibody. The same antibody was used with equal amounts of total cellular protein from the original samples to visualize total levels of Rac1.

Previous studies from our group have shown that the small GTPase Rac1 mediated the adhesion-dependency of TGF-β1-induced gene expression in PDAC cells [21]. To explore potential crosstalk of Rac1 with TGF-β1 antiproliferative signalling, we transfected PANC-1 cells with siRNA to Rac1 and assessed the effect on basal and exogenous TGF-β1-stimulated growth inhibition by [^3^H]-thymidine incorporation (Figure 2A, upper panel) and direct cell counting (Figure 2A, lower panel). As expected from its cell cycle activating function in other carcinoma cells, Rac1 depletion attenuated basal growth of cells cultured in normal growth medium (Figure 2A). Interestingly, however, in the same cells growth inhibition induced by exogenous TGF-β1 was clearly enhanced relative to unstimulated controls (indicated by the values below the bars in Figure 2). As shown by immunoblotting, the Rac1 siRNA, but not the irrelevant control, specifically diminished the level of both total Rac1 protein (Figure 2B, upper panel) and prevented the formation of active (GTP-bound) Rac1 in response to TGF-β1 stimulation (Figure 2B, lower panel). Similar data with respect to TGF-β1-induced growth inhibition were obtained for COLO 357 cells [Additional file 3 Figure S3]. These data show that depletion of Rac1 mimicks the effect of depletion of Smad2 on TGF-β1-mediated growth inhibition and led us to conclude that Rac1 antagonizes this cellular function of TGF-β1 in responsive PDAC cells.

### Specific inhibition of Rac1 activity potentiates growth inhibition induced by exogenous TGF-β1

**Figure 3:**
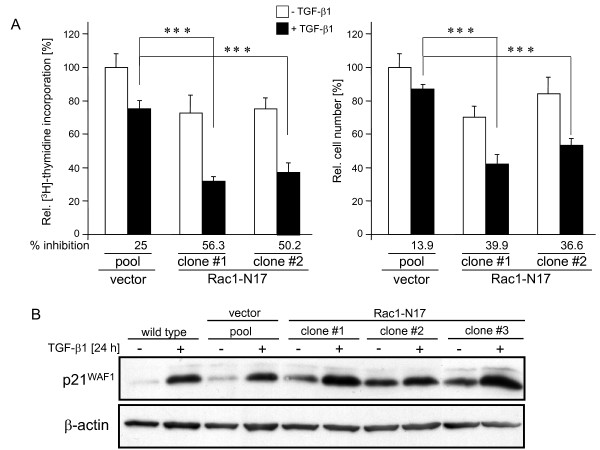
**Inhibition of Rac1 activity potentiates TGF-β1-induced growth inhibition and expression of p21^WAF1^**. (A) PANC-1 cells were retrovirally transduced in a stable fashion with dn Rac1 (N17 mutation) and several individual clones tested for their susceptibility to TGF-β1-mediated growth inhibition by [^3^H]-thymidine incorporation assay (left graph) and direct cell counting (right graph). **Data represent the mean ± standard deviation from six wells (thymidine incorporation) and 3 wells (cell numbers) processed in parallel**. Shown is a representative experiment each from three independent experiments. Asterisks, *p *< 0.001. (B) Immunoblot analysis of p21^WAF1 ^expression in three individual PANC-1-dn Rac1 clones and control cells stimulated or not with TGF-β1 for 24 h. β-actin was used as a loading control.

To scrutinize the role of Rac1 for pancreatic tumour cell proliferation and to evaluate whether the GTPase function of Rac1 was required for antagonizing TGF-β1-induced growth inhibition, we employed previously characterized PANC-1 clones stably expressing dn Rac1 (Rac1-N17) from a retroviral vector [21]. Several individual clones were found to have reduced basal growth and to respond to TGF-β1 with more pronounced growth inhibition when compared to empty vector controls (Figure 3A) or wild type cells (not shown) supporting our findings on siRNA-mediated suppression of *RAC1*. To exclude the possibility that enhanced apoptosis rather than growth inhibition accounted for lower cell numbers or reduced thymidine incorporation, we measured cell viability in cultures of PANC-1-dnRac1 stable clones and DNA fragmentation on PANC-1 cells transiently transfected with dn Rac1, or GADD45β as control (including both floating and adherent cells). Cell viability as assessed by trypanblue exclusion was low (< 5%) and was not significantly different between control and dn Rac1 expressing cells or between untreated and TGF-β-treated cells (data not shown). The observation that dn Rac1 lacked a proapoptotic effect was confirmed by a quantitative DNA fragmentation assay (Additional file 4 Figure S4). In contrast, ectopic expression of GADD45β, a Smad3-dependent TGF-β target gene [27] that can mediate TGF-β-induced apoptosis through p38 activation [28] (Additional file 4 Figure S4) sensitized PANC-1 cells to TGF-β1-induced DNA fragmentation. Together these experiments indicated that dn Rac1 suppressed proliferation rather than increasing apoptosis in both control and TGF-β1-treated cells. Next we investigated how Rac1 interacts with the cell cycle machinery to inhibit the TGF-β1 effect. A central mediator of TGF-β1-induced growth inhibition in PDAC is the cyclin-dependent kinase inhibitor p21^WAF1 ^[4,29-31]. Notably, in 3/3 PANC-1-dnRac1 clones analysed, basal and TGF-β1-induced levels of p21^WAF1 ^protein were clearly higher than in the wild type and vector controls as demonstrated by immunoblotting (Figure 3B), matching results from the Smad2 depletion experiments (Additional file 1 Figure S1A). Overall, these results indicate that inhibition of Rac1 GTPase activity, too, mimicked the effect of Smad2 knockdown on TGF-β1-dependent proliferation inhibition. We further conclude that in TGF-β1-responsive PDAC cells Rac1 activity promotes proliferation by partially antagonizing TGF-β1-mediated cytostasis via suppression of p21^WAF1 ^expression.

### Inhibition of RAC1 mimicks the effect of Smad2 silencing on basal and TGF-β1-induced cell motility

**Figure 4:**
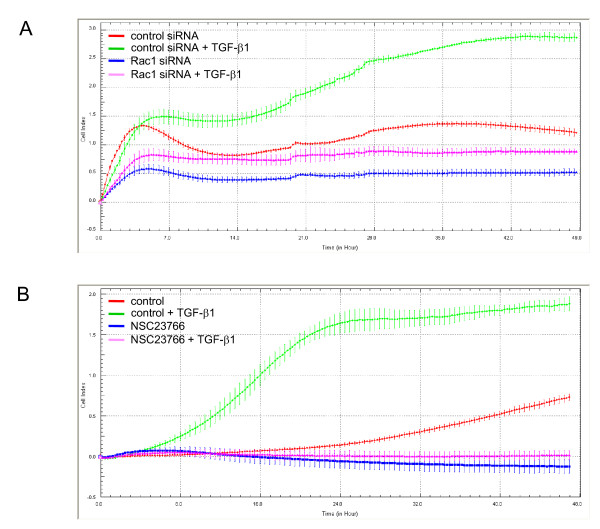
**Inhibition of Rac1 expression or activity mimics the effect of Smad2 silencing on basal and TGF-β1-induced cell motility**. **(**A) SiRNA-mediated silencing of Rac1 suppresses basal and TGF-β1-induced cell migration. PANC-1 cells were transfected twice with siRNAs to Rac1, or matched control siRNA, as described in the Method section and analysed for the cell's migratory response to TGF-β1 in RTCA cell migration assay. (B) Pharmacologic inhibition of Rac1 inhibits basal and TGF-β1-induced cell migration of PDAC cells. COLO 357 cells (60,000/well) were pretreated, or not, with 100 μM of the chemical Rac1 inhibitor NSC23766 for 1 h prior to analysis in the RTCA assay which was carried out in the presence or absence of 100 μM NSC23766. Data in (A) and (B) represent the mean ± standard deviation of four wells processed in parallel. Data are significant because of the large number of independently measured time points. Shown is a representative experiment chosen from a series of three independent experiments.

As shown above, siRNA-mediated knockdown experiments in PANC-1 cells suggested that Smad2 positively regulated TGF-β1-induced cell migration. To explore whether Rac1, too, promotes TGF-β1-induced motility, we transfected PANC-1 cells with Rac1 siRNA and assessed the effect on basal and TGF-β1-stimulated cell migration. Like Smad2 silencing, RAC1 silencing suppressed both basal and TGF-β1-induced cell migration but was more potent than Smad2 in this respect (Figure 4A). To confirm these results we, again, employed PANC-1 clones stably expressing dn Rac1 (the same clones utilized for assessment of growth inhibition above) and subjected them to real-time cellular migration (RTCA) assay. As expected, ectopic expression of dn Rac1, too, reduced basal migration and rendered the cells refractory to TGF-β1-stimulated cell motility when compared to empty vector and wild type controls (data not shown). Similar results in RTCA assays were obtained with both PANC-1 (data not shown) and COLO 357 cells (Figure 4B) treated with the chemical Rac1 inhibitor NSC23766. Taken together, the data clearly show that in PDAC cells basal migratory activity as well as the migratory response to TGF-β1 stimulation are strictly Rac1-dependent.

### Rac1 inhibition decreased TGF-β1/Smad2-dependent transcriptional activation but increased TGF-β1/Smad3-dependent transcriptional activation

**Figure 5:**
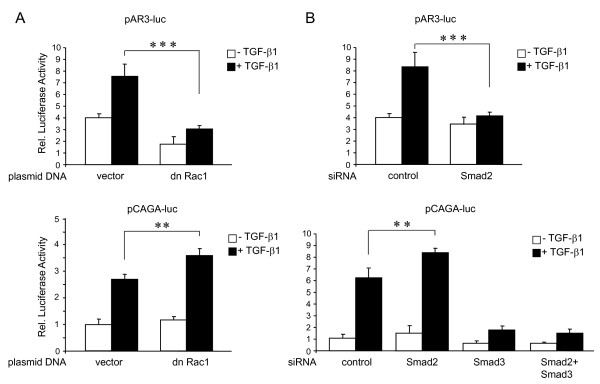
**Dn Rac1 abrogates Smad2-specific transcription but enhances Smad3-specifc transcription**. (A) PANC-1 cells were cotransfected with the Smad2-specific reporter plasmid pAR3-luc (+ FAST-1), or pCAGA-luc, and dn Rac1, or empty vector as indicated. **24 h after the start of transfection cells were stimulated with TGF-β1 for another 24 h and subjected to lysis**. Firefly luciferase activity was measured and normalized for *Renilla *luciferase activity. (B) As in (A) except that cells were transfected with either control siRNA, or Smad2, or Smad3 siRNAs (as indicated) instead of empty or dn Rac1 plasmid DNAs, as outlined in detail in the Method section. Twenty-four hours after the second round of transfection cells were stimulated for another 24 h with TGF-β1 followed by lysis and dual luciferase measurements. Shown are **data from one representative experiment (out of at least three performed in total), representing the normalized mean ± standard deviation of 6 wells processed in parallel**. **, *p *< 0.01; ***, *p *< 0.001.

Data presented so far indicate that depletion of Smad2 and inhibition of Rac1 in PANC-1 cells potentiated TGF-β1-induced growth inhibition and attenuated TGF-β1-induced cell motility, while depletion of Smad3 had the reciprocal outcome. This suggested a functional link in that Rac1 promotes activation of Smad2 while inhibiting activation of Smad3. To test this prediction more directly, we analysed in reporter gene assays how Rac1 would impact on Smad2 (and Smad3)-specific transcriptional activities, employing the reporter plasmids pAR3-luc (Smad2-dependent) and pCAGA-luc (Smad3-dependent). PANC-1 cells were transiently cotransfected with dn Rac1 and either pAR3-luc (+FAST-1) or pCAGA-luc and reporter gene activity was measured after 24 h of TGF-β1 stimulation. Notably, basal and exogenous TGF-β1-induced luciferase activity from pAR3-luc was suppressed by cotransfection of dn Rac1 relative to empty vector-transfected cells (Figure 5A, upper panel), while that from pCAGA-luc was enhanced albeit moderately (Figure 5A, lower panel). To verify whether changing the ratio of Smad2 and Smad3 would similarly affect transcriptional activation of pAR3-luc and pCAGA-luc by TGF-β1 we depeleted PANC-1 cells of the two R-Smads by siRNA transfection prior to TGF-β1 stimulation of reporter gene activity. As expected, depletion of Smad2 abrogated TGF-β1-induced transcriptional activity of pAR3-luc (Figure 5B, upper panel) but, notably, *enhanced *TGF-β1-induced activity of pCAGA-luc (Figure 5B, lower panel). In contrast, depletion of Smad3 as well as combined depletion of both Smad2 and Smad3 virtually abrogated pCAGA-luc activity (Figure 5B, lower panel), confirming the Smad3 dependency of the TGF-β1 effect on this reporter. These results are in favor of the idea that Rac1 differentially controls Smad2 and Smad3 activation and provide a molecular correlate to the effect of Rac1 on TGF-β1-controlled growth suppression.

### Inhibition of RAC1 abrogates TGF-β1-mediated phosphorylation of Smad2 but enhances that of Smad3

**Figure 6:**
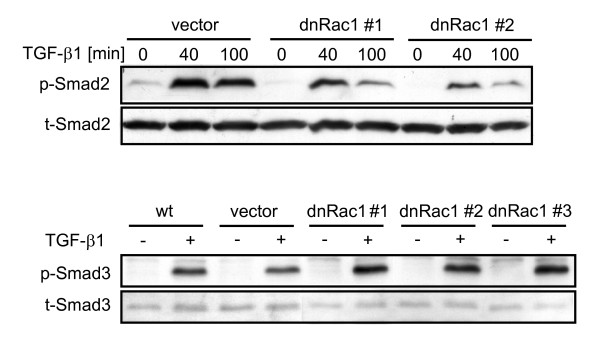
**Inhibition of Rac1 differentially affects TGF-β1-mediated C-terminal domain phosphorylation of Smad2 and Smad3**. PANC-1-dn Rac1 cells used in Fig. 3 were analysed along with wild type (wt) cells and empty vector (vector)-transduced control cells by immunoblotting for their ability to phosphorylate Smad2 (p-Smad2, upper panel) and Smad3 (p-Smad3, lower panel) in response to TGF-β1 stimulation (Smad3: 1 h, Smad2: as indicated in the figure). Total Smad levels (t-Smad) were detected as loading controls.

The results presented above provided evidence that Rac1 may directly control the activation of both R-Smads in PDAC cells. More specifically, we hypothesized that Rac1 alters the activation state of Smad2 and Smad3 by modifying their phosphorylation on serine residues located at the C-terminus (Smad2: Ser465/467, Smad3: Ser423/425). To test this assumption, we first analysed whether dn Rac1 inhibition can alter TGF-β1-mediated activation of Smad2. Notably, TGF-β1 stimulated (and unstimulated) p-Smad2 was severely reduced in dn Rac1 expressing PANC-1 clones (Figure 6, upper panel: two representative clones are shown). In order to rule out clonal artefacts, we transiently co-transfected PANC-1 cells with FLAG-tagged Smad2 along with either HA-tagged FRNK or MYC-tagged dn Rac1 and evaluated levels of p-Smad2 following TGF-β1 stimulation. As seen in the stable transfectants, dn Rac1 but not FRNK, a kinase-deficient mutant and endogenous inhibitor of p125^FAK ^[32], abolished phosphorylation of Smad2 [data not shown] and thus attest to the Rac1-dependency of TGF-β1-induced Smad2 activation in PANC-1 cells. Inhibition of TGF-β1-induced p-Smad2 was also seen in COLO 357 cells following Rac1 inhibition with NSC23766 (Additional file 5 Figure S5). Since Rac1 inhibition enhanced TGF-β1-mediated growth inhibition (see Figures 2 and 3) and Smad3-dependent transcriptional activity (see Figure 5), we evaluated whether inhibition of Rac1 activity in PANC-1 cells would also affect Smad3 activation by the TβRI/ALK5 kinase. Interestingly, stable expression of dn Rac1 was associated with a slight increase rather than a decrease in p-Smad3 levels in 3 individual clones compared to wild type and empty vector controls (Figure 6, lower panel). These data show that Rac1 differentially controls the activation of Smad2 and Smad3 through phosphorylation at the C-terminus in a way that corresponds well with the differential functional outcomes of direct inhibition of both R-Smads. This further supports our hypothesis that Rac1 promotes Smad2-mediated TGF-β1 responses, e. g. chemokinesis, while suppressing Smad3-dependent responses, like growth inhibition.

### The growth-inhibitory effect afforded by Rac1 inhibition and the Smad2 activating function of constitutively active Rac1 are reduced upon disruption of autocrine TGF-β signalling

**Figure 7:**
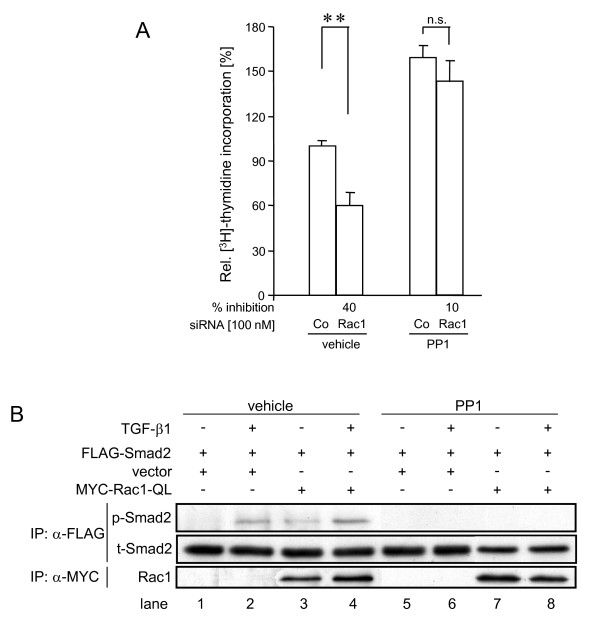
**Blocking autocrine TGF-β signalling renders PANC-1 cells resistant towards the growth-suppressive effect of Rac1 inhibition**. (A) PANC-1 cells were transfected with Rac1 or control (Co) siRNA as outlined in the legend to figure 1. Fourty-eight hours later cells were treated with PP1 (10 μM) or vehicle for 24 h and subjected to [^3^H]-thymidine incorporation assay. Data represent the mean ± standard deviation from six wells processed in parallel. **, *p *< 0.01; ***, *p *< 0.001; n.s., not significant. (B) PANC-1 cells were transiently cotransfected with FLAG-tagged Smad2 and a MYC-tagged version of ca Rac1 (Q61L) as indicated over the figure. 24 h after transfection cells were stimulated or not with TGF-β1 for 1 h in the absence or presence of 10 μM PP1. Following lysis and anti-FLAG and anti-MYC IPs, samples were analysed by sequential immunoblotting for phospho- (p-) and total (t-) Smad2 as well as Rac1.

As seen in Figure 2, 3, and 4, Rac1 inhibition by both siRNA transfection and dn interference lowered proliferation and cell migration not only in TGF-β1-stimulated but also in the absence of exogenous TGF-β1, suggesting that both growth and motility are partially controlled in a TGF-β1-independent manner. However, the observation that PANC-1 cells secrete biologically active TGF-β1 in vitro [33] may mean that cells could inhibit their growth and stimulate their migration in an autocrine fashion, and, consequently, that Rac1 "protects" cells from autocrine growth inhibition but at the same time ensures autocrine stimulation of cell migration. We investigated this possibility for the growth promoting role of Rac1. To do this we used PP1, a small molecule compound that has recently been shown in mammary epithelial cells [34] and in PANC-1 cells [35] to potently inhibit the kinase activity of TβRI/ALK5, to suppress TGF-β1-induced phosphorylation of Smad2 and Smad3 and EMT [35]. In addition, we have demonstrated that PP1 dose-dependently relieved the growth suppressive effect of TGF-β1 in a Src-unrelated fashion [35]. To determine whether the autocrine TGF-β growth-inhibitory loop was subject to regulation by Rac1, we evaluated the effect of Rac1 depletion on proliferative activity upon silenced autocrine TGF-β signalling under PP1 treatment. As shown in Figure 7A, PP1 increased the DNA synthesis (as a measure of the proliferative activity) in PANC-1 cells and, importantly, decreased the growth-inhibitory effect of Rac1 siRNA when compared to vehicle controls.

We further reasoned that if TGF-β autostimulation was permanently operating in cultures of PANC-1 cells (as suggested by clearly detectable levels of p-Smad2/3 in unstimulated cells, see Additional file 1 Figure S1B, and Figure 6) then ectopic expression of a ca mutant of Rac1 (Q61L) should be able to stimulate p-Smad2 even in the absence of exogenous TGF-β1. This assumption was tested in transient cotransfection/immunoprecipitation assays. Here, ca Rac1 was able to enhance the amount of p-Smad2 over empty vector control samples in the absence of added TGF-β1 and PP1 (Figure 7B, lane 3), but was unable to do so in the presence of PP1 (Figure 7B, lane 7). Together, these data strongly suggest that Rac1 modulates Smad signalling in response to both exogenous and autocrine TGF-β(s) signalling.

## Discussion

In this study we initially presented evidence that TGF-β1-induced growth inhibition and cell migration in PDAC cells were differentially and selectively controlled by Smad3 and Smad2, respectively. Knockdown of Smad3 but not Smad2 relieved TGF-β1-induced growth inhibition, indicating that this response was Smad3-dependent, an observation made previously in various other cell types including PANC-1 cells [9,36,25]. In contrast, knockdown of Smad2 decreased the TGF-β1-driven motility of PDAC cells revealing cell migration (or more precisely chemokinesis) to be a Smad2-specific response. This is in line with the demonstration of a crucial role of Smad2 in regulating keratinocyte migration during wound healing [37]. We went on to describe first-time observations, namely that the effects of Smad2 depletion on TGF-β1-mediated growth inhibition and cell migration were largely mimicked by inhibition of Rac1 expression (via siRNA knockdown) or activity (via ectopic expression of a dn Rac1 mutant), or pharmacologic inhibition (via NSC23766), together suggesting a functional link between both proteins. We subsequently confirmed this assumption by showing that Rac1 inhibition abrogated TGF-β1-induced Smad2-specific C-terminal phosphorylation and transcriptional activity but increased TGF-β1-mediated p21^WAF1 ^expression. Another interesting and novel observation of this study was the mutual amplification of effects such that knockdown of Smad2 or inhibition of Rac1 (without direct modulation of Smad3) enhanced growth inhibition, Smad3-specific transcriptional activity, and C-terminal phosphorylation of Smad3, while knockdown of Smad3 (without direct modulation of Smad2) enhanced both Smad2-specific responses such as cellular migration (this study) and Smad2 phosphorylation by TGF-β [25]. This suggested functional antagonism between the two R-Smads and that the ratio of Smad3 to Smad2 determines the ultimate outcome of the TGF-β response as demonstrated previously for TGF-β-induced growth inhibition in PANC-1 cells [25].

The decreases in basal proliferation of PANC-1 and COLO 357 cells following Rac1 inhibition may be largely due to disruption of promitogenic growth factor signalling. PDAC cells, e.g. PANC-1 cells, are well known to autostimulate their proliferation in culture via secretion of EGF. Consequently, both the tyrosine kinase inhibitor tyrphostin AG1478 and the ERK inhibitor U0126 dramatically inhibited PANC-1 cell proliferation (H. U., unpublished data). The intimate relationship between the TGF-β and EGF-R pathways in growth regulation of carcinoma cells is also evident from studies showing that TGF-β1 can suppress PDAC cell proliferation by repressing EGF-R-induced ERK activation [38] and that EGF signalling, in turn, is permissive for regulation of gene expression and growth suppression by TGF-β1 [39]. Previous observations of TGF-β1 secretion in vitro [33], and suppression of "basal" p-Smad2/3 levels and *BGN *mRNA upon ALK5 inhibition [21,23,40] clearly suggested that PANC-1 cells may also exhibit autocrine TGF-β growth inhibition. Previous studies in breast cancer cells have shown that cell cycle progression/inhibition is subject to regulation by autocrine TGF-β [41,42]. In order to block autocrine TGF-β signalling we used PP1, which in PDAC cells effectively blunted growth inhibition induced by exogenously added and autocrine TGF-β's (Figure 7). Importantly, in the presence of PP1 siRNA-mediated Rac1 depletion resulted in much less growth inhibition than in control transfected cells with functional TGF-β/Smad signalling. Hence, reduced DNA synthesis in cells with low Rac1 activity (and not exposed to exogenous TGF-β1) may, at least in part, be explained by increased susceptibility to autocrine growth inhibition by TGF-β's. Similar observations (an increase in growth suppression even in the absence of exogenous TGF-β) were made by Kim and coworkers [25] upon depletion of Smad2 in PANC-1 cells and these authors showed that this response disappeared in the presence of neutralizing anti-TGF-β antibody. These results perfectly match our data on the sensitization to autocrine TGF-β responses obtained through pharmacologic inhibition of ALK5 and further support our hypothesis of Rac1-mediated control of Smad2 activation.

Interestingly, the decrease in basal and TGF-β1-induced growth upon dn Rac1 expression was accompanied by a respective increase in expression of *p21^WAF1^*. In line with these results, Rac1 activity was both necessary and sufficient for suppression of p21^WAF1 ^in prostate cancer cells [19].

As discussed above, the decreases in basal proliferation following Rac1 inhibition may involve both disruption of promitogenic growth factor signalling and loss of protection from autocrine TGF-β-mediated growth inhibition as a consequence of the shift from p-Smad2 to p-Smad3 signalling. Similarly, as the inhibition of Rac1 was much more effective in suppressing basal and TGF-β1-induced cell migration than was the inhibition of Smad2 expression (compare Figures 2 and 5), Rac1 is likely to control cell motility, too, in part in an autocrine TGF-β-dependent (and TGF-β-independent) fashion.

There is now ample evidence that Smad2 and Smad3 have distinct functional and non-overlapping roles in TGF-β signalling [9] implying that intracellular factors which control the relative activation state of Smad2 versus Smad3 signalling have a central role in determining the final outcome of the TGF-β response. Here, we showed that PANC-1 cells responded to inhibition of Rac1 with a pronounced decrease in TGF-β1-mediated p-Smad2 and a slight increase in p-Smad3. In agreement with these data, dn Rac1 expression not only decreased Smad2-specific transcriptional activity (on pAR3-luc) but enhanced general Smad3-specific transcriptional activity (on pCAGA-luc). Moreover, dn Rac1 also increased *p21^WAF1 ^*protein expression which is in line with data showing that *p21^WAF1 ^*was transcriptionally induced by TGF-β in a Smad3-dependent manner in pancreatic, hepatic and skin cells [30,18,43]. However, TGF-β-induced transcription of another reporter gene (p3TP-lux) in HepG2 cells was effectively inhibited by Rac1-N17 expression [20] which might be explained by the fact that this plasmid is partially responsive to non-Smad (e.g. p38 MAPK) signalling. With respect to the functional antagonism observed, a likely explanation is that Smad2 and Smad3 compete with each other either i) for binding to TβRI/ALK5, ii) capture of Smad4 in the cytoplasm, or iii) recruitment of transcriptional corepressors to SBEs in the nucleus, the latter of which is normally performed by Smad2 [1]. As a consequence, a reduction in Smad2 expression or activation would increase the ability of Smad4 to bind Smad3 on the SBEs of target gene promoters. In agreement with this possibility are experiments in PANC-1 cells, in which direct silencing of Smad2 via siRNA transfection did not only augment TGF-β1-induced Smad3 phosphorylation [25], p21^WAF1 ^expression and growth inhibition (Additional file 1 Figure S1 and Ref. [25]), but also potentiated TGF-β1-induction of Smad3-regulated genes such as *MMP2 *and *BGN *(see Figure 1). Indirect evidence that the endogenous ratio of Smad2 and Smad3 determines the quality of the TGF-β response was observed in Hep3B cells, in which the expression of Smad3-Smad4-dependent TGF-β target genes was further enhanced after selective knockdown of *SMAD2 *[44], and in mouse keratinocytes, in which Smad2 loss led to a significant increase in Smad3-Smad4 binding to the promoter of the transcription factor Snail, Snail upregulation, and EMT [45]. Indirect evidence that competition can be mutual comes from a study with Smad2 and Smad3-deficient fibroblasts, in which activation of the pAR3-luc reporter, though strongly suppressed in Smad2-deficient fibroblasts, was *enhanced *in Smad3-null cells [46]. Regarding the intracellular site of competition (see above) our data favour Smad recruitment or binding to ALK5 since dn Rac1 stimulated a shift from p-Smad2 to p-Smad3.

As mentioned above, Rac1 has been found to be overexpressed in PDAC [17] along with high activity of Vav1 [11]. Hyperactive Rac1 could therefore increase basal growth through its (TGF-β/Smad-independent) growth promoting effect and, at the same time, protect tumour cells, which have not yet accumulated inactivating mutations in the TGF-β pathway, from exaggerated growth restraints by TGF-β. More specifically, Rac1 aids cancer cells to more efficiently antagonize TGF-β1/Smad3-mediated growth inhibition via its ability to promote Smad2 activation. Interestingly, hyperactive Ras (present in both PANC-1 and COLO 357 cells) has been shown, like Rac1, to suppress ALK5-mediated Smad3 phosphorylation and growth inhibition [47]. Oncogenic Ras-induced transformation can lead to the production of superoxide through one or more pathways involving NAD(P)H oxidase(s)/Nox1 and Rac1 [48]. In this way Rac1 may act as a mediator of Ras-induced cell cycle progression independent of MAPK and JNK and may contribute to the unchecked proliferation of Ras-transformed cells [48]. Notably, preliminary data from our laboratory indicate that Rac1 acts through ROS and NAD(P)H oxidase to promote Smad2 phosphorylation (H. U., unpublished observation).

The mechanism described here for Rac1 differs from the previously described ones in that it reciprocally targets Smad2 and Smad3 at the posttranscriptional level. It is widely appreciated that Rac1 acts in a prooncogenic fashion during later stages of tumour progression by promoting migration, invasion, and metastasis [13,14]. In addition to fundamental differences in the mechanism of Smad2 and Smad3 activation by TGF-β1, at least in PDAC cells, our study reveals that Rac1 may drive tumourigenesis in carcinoma cells with a still intact TGF-β/Smad pathway by favouring resistance to TGF-β1-mediated growth inhibition and by increasing TGF-β1-induced cell migration at the R-Smad epigenetic level.

## Conclusions

In malignant PDAC cells with a functional TGF-β signalling pathway Rac1 antagonizes the TGF-β1 cytostatic response and enhances cell migration by differentially regulating Smad2 *and *Smad3 activation. Thus, Rac1 may be employed by cells as a switch to fine-tune Smad2 *versus *Smad3-dependent TGF-β1 responses. This study reveals that Rac1 is prooncogenic in that it can alter TGF-β signalling at the R-Smad level from a tumour-suppressive towards a tumour-promoting outcome.

## Methods

### Antibodies and reagents

TGF-β1 was purchased from R&D Systems (Wiesbaden, Germany). The antibodies and their suppliers were: Rac1, p21^WAF1^: BD Transduction Laboratories (Heidelberg, Germany); phospho-Smad2 (Ser465/467), phospho-Smad3 (Ser423/425)/Smad1(Ser463/465), HSP90, MYC-Tag (clone 9B11): Cell Signalling Technology (Heidelberg, Germany); Smad2: Zymed (Berlin, Germany); FAK (C-20), Smad2/3 (E-20): Santa Cruz Biotechnology (Heidelberg, Germany); β-actin, FLAG (M2): Sigma (Deisenhofen, Germany); HA (12CA5): Roche Diagnostics (Mannheim, Germany), active Rac1: NewEast Biosciences (Malvern, USA). PP1 analog, the Smad3 inhibitor SIS3, and the Rac1 inhibitor NSC23766 were purchased from Calbiochem/Merck (Darmstadt, Germany). Pharmacological inhibitors were added to cells 30 min before the addition of TGF-β1 which was used at 5 ng/ml for both PANC-1 and COLO 357 cells.

### Cell lines and cell culture

Maintenance of the human PDAC cell lines PANC-1 and COLO 357 was described earlier [23]. PANC-1 cells stably transduced with dn Rac1 retroviral vectors were cultured in the presence of 2.5 μg/ml puromycin (Sigma).

### RNA isolation and RT-PCR analysis

Total RNA from PANC-1 cells was isolated with peqGOLD RNAPure (Peqlab, Erlangen, Germany) and reverse transcribed using Superscript II Reverse Transcriptase (Invitrogen). The primer sequences for BGN, β-actin, MMP-2, and TATA box binding protein (TBP) were given earlier [7,23]. The mRNA expression was quantified by quantitative real-time RT-PCR (qPCR) on an I-Cycler (Bio-Rad, München, Germany) with I-Cycler software (Bio-Rad). SYBR green was used for detection of amplification products. All values for BGN and MMP-2 mRNA concentrations were normalized to those for β-actin and TBP-specific transcripts in the same sample to account for small differences in cDNA input.

### Construction of vectors and retroviral infection

The construction of a retroviral vector (pBABEpuro) for human dn Rac1 (T17N mutation) [21] and of pcDNA3-based expression vectors for FLAG-tagged Smad2 and GADD45β [40] was described previously. A cDNA insert of a MYC-tagged version of dn Rac1 was released from the pRK5-MYC vector and subcloned in pcDNA3.

### Transient transfections of expression vectors and siRNAs and reporter gene assays

For transient transfections followed by immunoprecipitation (IP), PANC-1 cells were seeded at a density of 2 × 10^4 ^cells/cm^2 ^in 6-cm plates on day 1, and on day 2 were co-transfected serum-free with Lipofectamine Plus (Invitrogen) according to the manufacturer's instructions with FLAG-tagged Smad2 in combination with either empty pcDNA3 vector, HA-tagged FRNK, MYC-tagged dn Rac1 (T17N), or MYC-tagged ca Rac1 (Q61L) as indicated in the legend to Figure 7. Following removal of the transfection solution and a recovery period of 24 h in normal growth medium, cells were stimulated with TGF-β1 for 1 h. The transfected cells were then lysed in IP buffer (20 mM Tris, pH 7.5, 150 mM NaCl, 1 mM EDTA, 1 mM EGTA, 1% Triton X-100, 2.5 mM sodium pyrophosphate, 1 mM β-glycerolphosphate, 1 mM Na_3_VO_4_, 1 μg/ml leupeptin, 1 mM PMSF) and processed for anti-FLAG, anti-HA, and anti-MYC immunoprecipitation and immunoblotting (see below). SiRNAs specific for Rac1 (ON-TARGETplus SMARTpool, a mixture of four prevalidated siRNAs) and matched negative control (non-target control SMARTpool) were purchased from Thermo Scientific Dharmacon (Epsom, UK), while prevalidated siRNAs to Smad2 and Smad3 as well as matched control were from Qiagen (Hilden, Germany) [33]. Rac1, Smad2/3, and negative control siRNAs were transfected twice on two consecutive days with either Lipofectamine 2000 or Lipofectamine RNAiMax (PANC-1) and HiperFect (COLO 357) (all from Invitrogen) according to the supplier's recommendations. For reporter gene assays, cells were seeded in 96-well plates and were co-transfected on the next day serum-free with either Lipofectamine Plus or Lipofectamine 2000 (Invitrogen) with various cDNAs (in pcDNA3) at an equal molar ratio together with dn Rac1 and either pAR3-luc+FAST-1, or pCAGA-luc, along with the *Renilla *luciferase encoding vector pRL-TK (Promega, Mannheim, Germany). Each well received the same total amount of DNA (0.2 μg) and empty vector was added as needed. Following transfection and TGF-β1 stimulation, luciferase activities were determined with the Dual Luciferase Assay System (Promega). Pilot experiments with pCAGA-luc and increasing concentrations (5, 25, and 100 ng/well) of dn Rac1-pcDNA3 DNA indicated that the effect of dn Rac1 was dose-dependent (data not shown). In case of combined siRNA/plasmid DNA transfections PANC-1 cells underwent a first round of transfection with siRNA alone (on day 1 after seeding) and Lipofectamine RNAiMax, followed by a second round with siRNA plus plasmid DNA and Lipofectamine 2000 (on day 2). In all reporter gene assays the data were derived from 6-8 wells processed in parallel and corrected for transfection efficiency with *Renilla *luciferase activity.

### Immunoprecipitation and immunoblot analysis

Epitope-tagged proteins were immunoprecipitated from cellular lysates with anti-FLAG, anti-HA, or anti-MYC antibodies and Protein A Sepharose Fast Flow (Amersham Biosciences, Freiburg, Germany) or protein G Plus Sepharose (Santa Cruz Biotechnology) according to the protocol provided by the supplier, and subsequently analyzed by SDS-PAGE and immunoblotting as described in detail earlier [23].

### Proliferation and apoptosis assays

Cell counting of was performed with Cedex XS cell analysis system (Roche Diagnostics, Mannheim, Germany) according to the instruction manual. The methyl-[^3^H]-thymidine incorporation assay was essentially carried out as described previously [7,23]. Twenty-four hours after transient transfection with expression plasmids for dn Rac1 or GADD45β, a JAM DNA fragmentation assay was performed as outlined in detail earlier [49]. Briefly, transfected PANC-1 cells were trypsinized and reseeded at a density of 1-2 × 10^4 ^cells/well into 96-well flat bottom plates, allowed to adhere overnight and labelled with [^3^H]-thymidine (370 KBq/μl) for 4 h. Subsequently, non-incorporated radioactivity was removed by washing the cells with PBS. Following incubation with TGF-β1 in normal growth medium for 24 h, cells were harvested by vacuum aspiration on glass fiber filters. Dried filters were counted into a liquid scintillation counter (Wallac, Switzerland). The percentage of specific DNA fragmentation, indicative of apoptosis, was calculated as: % viability = (*E/S*) × 100, where *E *(experimental) is cpm of retained DNA in the presence of TGF-β1 and *S *(spontaneous) is cpm of retained DNA in the absence of TGF-β1.

### Measurement of cell migration

Using the xCELLigence DP device from Roche Diagnostics real-time measurements of cell migration on wild type or transfected PANC-1 and COLO 357 cells were performed. 60,000-90,000 cells were seeded per well in CIM-Plates 16 (Roche Diagnostics). Prior to cell seeding the underside of the wells was coated with collagen I (400 μg/ml, Sigma, Deisenhofen, Germany) which was chosen since it represents the major matrix protein in PDAC tissue. TGF-β1 (and in some experiments pharmacologic inhibitors) were added to both lower and upper wells at the same concentration. The RTCA assay was performed as detailled by Roche Diagnostics in the instruction manual. In those experiments in which cells underwent transfection they were processed to enter the assay 24-48 hrs after the second round of transfection. In experiments involving small molecule inhibitors, cells were pretreated for 1 h before the addition of TGF-β1. Data acquisition and analysis were performed with the RTCA software (version 1.2, Roche Diagnostics) over a period of 48 h.

### Statistical analysis

Statistical significance was calculated using the unpaired student's *t*-test. Data were considered significant at *p *< 0.05. Calculated levels of significance were *p *< 0.05 (*), *p *< 0.01 (**), and *p *< 0.001 (***).

## List of abbreviations

ALK5: activin receptor-like kinase 5; ca: constitutively active; BGN: biglycan; EGF(-R): epidermal growth factor(-receptor); EMT: epithelial-to-mesenchymal transition; ERK: extracellular signal-regulated kinase; FRNK: FAK-related non-kinase; GEF: guanine exchange factor; IP: immunoprecipitation; kd: kinase-deficient; MAPK: mitogen-activated protein kinase; MMP-2: matrix metalloprotease-2; NAD(P)H: nucleotide adenine diphosphate hydrogenase; PAI-1: plasminogen activator inhibitor-1; PDAC: pancreatic ductal adenocarcinoma; PI3-K: phosphoinositol-3-kinase; ROS: reactive oxygen species; R-Smad: receptor regulated Smad; SBE: Smad binding element; TBP: TATA box binding protein; TGF-β; transforming growth factor-β;

## Competing interests

The authors declare that they have no competing interests.

## Authors' contributions

HU, SG and SS performed the experiments. FG and HL contributed to the interpretation and discussion of the results. Both HU and FF are the principal investigators and were involved in the conceptualization and discussion of the manuscript. HU wrote the manuscript. All authors read and approved the final version of the manuscript.

## Supplementary Material

Additional file 1 **Figure S1**. Effect of siRNA-mediated silencing of Smad2 and Smad3 on TGF-β1-induced growth suppression in PANC-1 cells. [^3^H]-thymidine incorporation assay of PANC-1 cells depleted of Smad2 or Smad3 by siRNA transfection. Click here for file

Additional file 2 **Figure S2**. Effect of Smad3 inhibition and Smad2 depletion on TGF-β1-induced chemokinesis in COLO 357 cells as measured with the RTCA real-time cell migration assay. (Figure S1) Migratory response of TGF-β1-treated COLO 357 cells in the absence or presence of a pharmacologic Smad3 inhibitor and (Figure S2) after siRNA-mediated depletion of Smad2. Click here for file

Additional file 3 **Figure S3**. SiRNA-mediated depletion of Rac1 decreases basal proliferation and enhances TGF-β1-induced growth suppression. Proliferation assay of TGF-β1-treated COLO 357 cells transiently transfected with Rac1 siRNA. Click here for file

Additional file 4 **Figure S4**. Reduced thymidine incorporation by Rac1 suppression is the result of reduced proliferation rather than increased apoptosis. Apoptosis assay of TGF-β1-treated PANC-1 cells transiently transfected with dn Rac1. Click here for file

Additional file 5 **Figure S5**. Immunoblot analysis of TGF-β1-mediated phosphorylation of (endogenous) Smad2 in COLO 357 cells in the absence or presence of the pharmacologic Rac1 inhibitor NSC23766. Click here for file
