# Supplementary material for: Differential roles of Smad2 and Smad3 in the regulation of TGF-β1-mediated growth inhibition and cell migration in pancreatic ductal adenocarcinoma cells: control by Rac1
Source: Mol Cancer. 2011 May 30;10:67. doi: 10.1186/1476-4598-10-67 (PMC3112431; doi:10.1186/1476-4598-10-67)
Supplement: Additional file 1 — Figure S1. Effect of siRNA-mediated silencing of Smad2 and Smad3 on TGF-β1-induced growth suppression in PANC-1 cells. [3H]-thymidine incorporation assay of PANC-1 cells depleted of Smad2 or Smad3 by siRNA transfection. [file 1476-4598-10-67-S1.PDF]

A

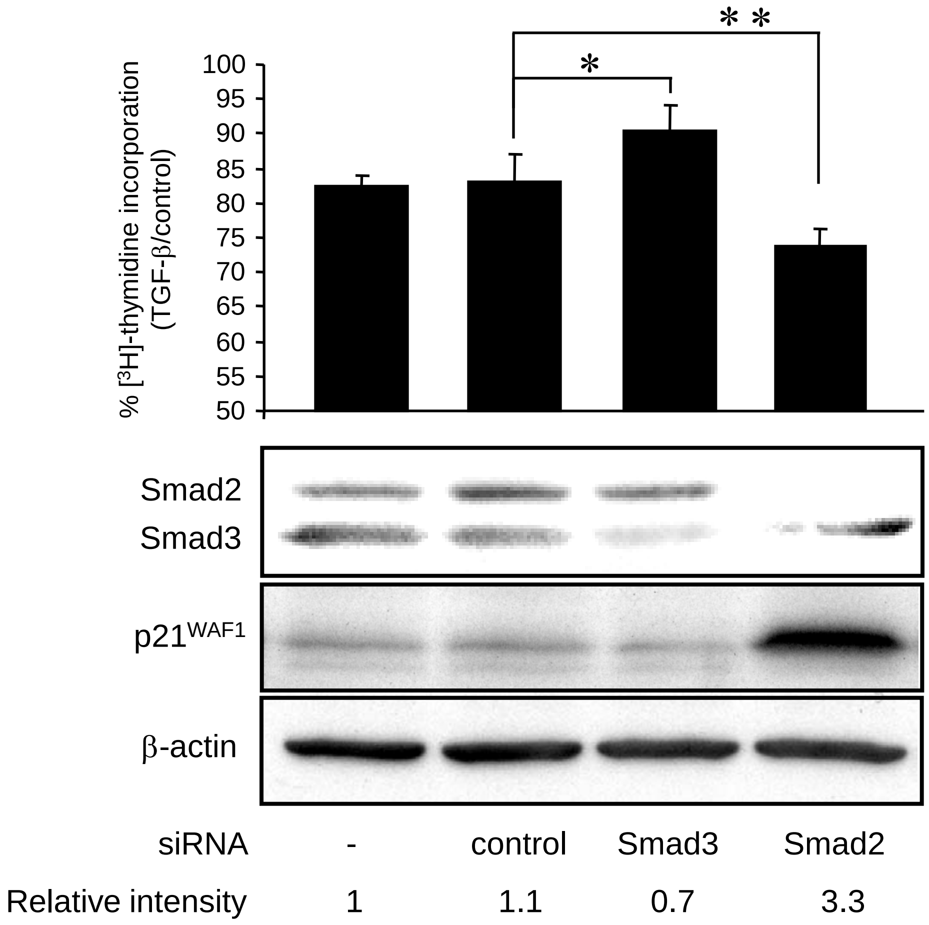

Legend to Figure S1A: **Effect of siRNA-mediated silencing of Smad2 and Smad3 on TGF-β1-induced growth suppression in PANC-1 cells.** PANC-1 cells were transfected with transfection agent alone (-), control siRNA, or siRNAs against Smad3 or Smad2. 72 h after transfection PANC-1 cells were seeded into 96-well plates (in normal growth medium) and remained unstimulated or were stimulated on the next day with TGF-β1 (5 ng/ml) for another 24 h. [^{3}H]-thymidine was added to the cells for the last 4 h of the incubation period. Data (means±standard deviations from six replicates) are taken from one out of two independent experiments and are depicted as the percentage of growth inhibition of TGF-β1-treated relative to control siRNA-transfected cells (set arbitrarily at 100%). ✶, *p*<0.05; ✶✶, *p*<0.01. Note that the scale of the ordinate starts at 50%.
Lower panel, PANC-1 transfectants were lysed and subjected to immunoblot analysis to verifyspecificity and selectivity of the siRNAs for the respective Smads.
The same blot was successively probed for Smad2/3, $p21^{WAF1}$, and β-actin to control for equal loading. Relative band intensities were determined by densitometric scanning of underexposed blots using NIH image software. Control immunoblots indicated that the expression patterns for Smad2, Smad3 and $p21^{WAF1}$ were stable over the assay period (data not shown).

B

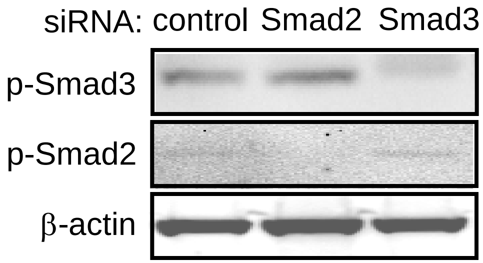

Legend to Figure S1B: As in (A) except that the PANC-1 cellular extracts were probed for phospho-Smad3 (p-Smad3) and phospho-Smad2 (p-Smad2).
