# Supplementary material for: Differential roles of Smad2 and Smad3 in the regulation of TGF-β1-mediated growth inhibition and cell migration in pancreatic ductal adenocarcinoma cells: control by Rac1
Source: Mol Cancer. 2011 May 30;10:67. doi: 10.1186/1476-4598-10-67 (PMC3112431; doi:10.1186/1476-4598-10-67)
Supplement: Additional file 2 — Figure S2. Effect of Smad3 inhibition and Smad2 depletion on TGF-β1-induced chemokinesis in COLO 357 cells as measured with the RTCA real-time cell migration assay. (Figure S1) Migratory response of TGF-β1-treated COLO 357 cells in the absence or presence of a pharmacologic Smad3 inhibitor and (Figure S2) after siRNA-mediated depletion of Smad2. [file 1476-4598-10-67-S2.PDF]

A

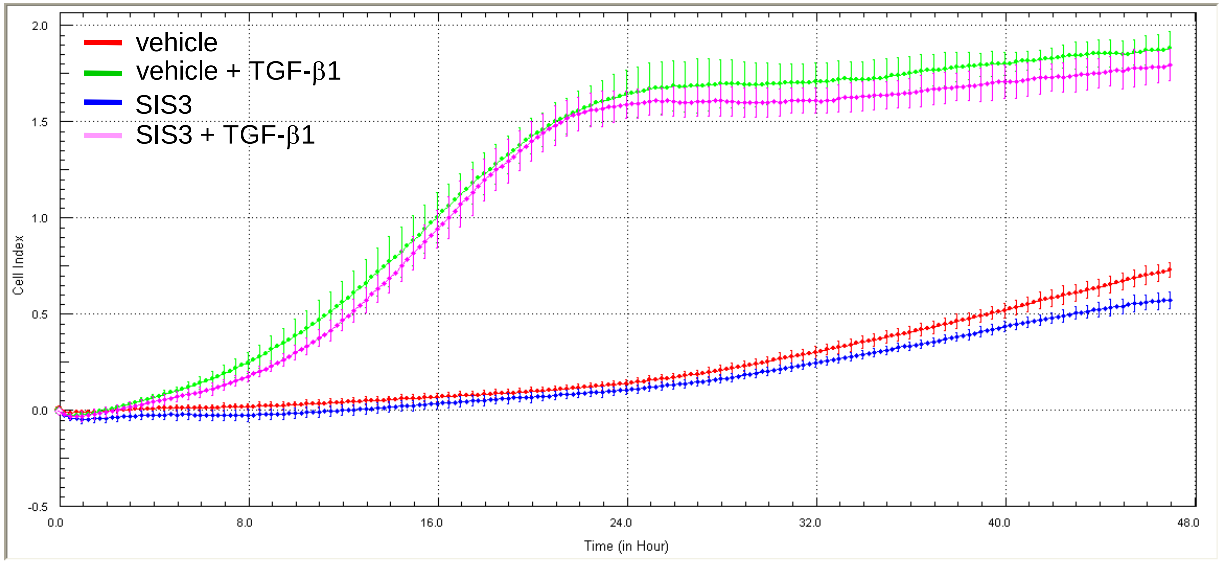

Legend to Figure S2A: **Effect of Smad3 inhibition on TGF-β1-induced cell migration in COLO 357 cells.**
A, COLO 357 cells (60,000 cells/well) were subjected to xCELLigence real-time cell migration assay in the presence or absence of TGF-β1 and SIS3, a pharmacologic Smad3 inhibitor that has been shown not to cross-inhibit Smad2. Data represent the means ± standard deviations from quadruplicate wells. One out of two experiments is shown. Data points of the green and pink curves were not significantly different.

B

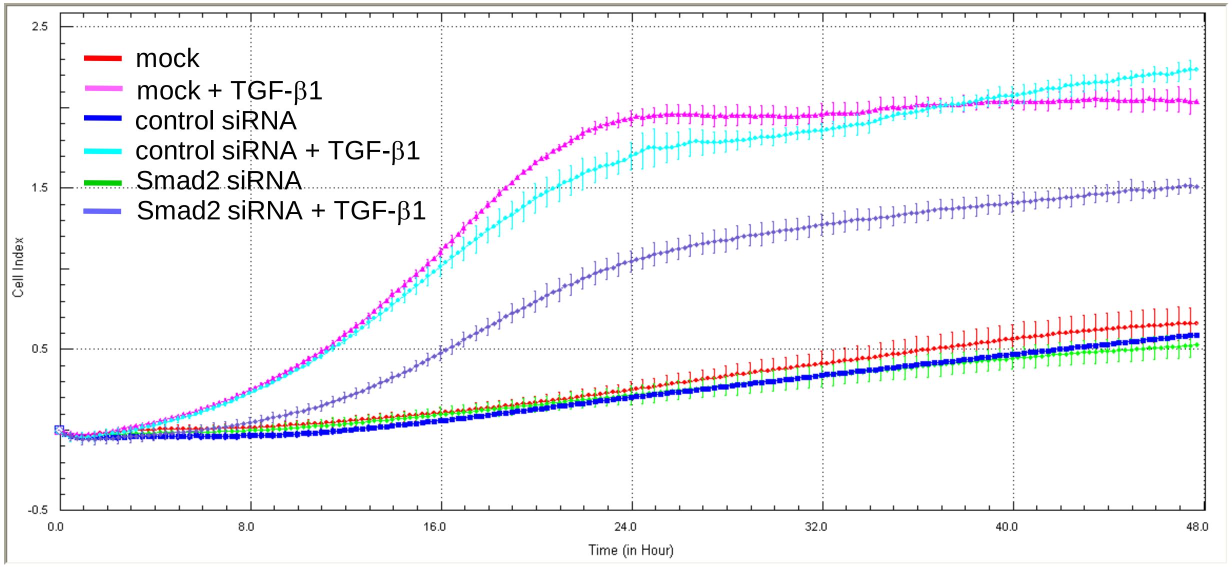

Legend to Figure S2B: **Smad2 depletion inhibits TGF-β1-induced cell migration in COLO 357 cells.**
A, COLO 357 cells (60,000 cells/well) were transiently transfected with control siRNA or Smad2-specific siRNA and subjected to xCELLigence real-time cell migration assay in the presence or absence of TGF-β1.
Data represent the means ± standard deviations from quadruplicate wells. One representative experiment from three experiments is shown. Data points of the Smad2 siRNA transfected cells were significantly different from those of the mock and control siRNA transfected cells.
