# Supplementary material for: Differential roles of Smad2 and Smad3 in the regulation of TGF-β1-mediated growth inhibition and cell migration in pancreatic ductal adenocarcinoma cells: control by Rac1
Source: Mol Cancer. 2011 May 30;10:67. doi: 10.1186/1476-4598-10-67 (PMC3112431; doi:10.1186/1476-4598-10-67)
Supplement: Additional file 3 — Figure S3. SiRNA-mediated depletion of Rac1 decreases basal proliferation and enhances TGF-β1-induced growth suppression. Proliferation assay of TGF-β1-treated COLO 357 cells transiently transfected with Rac1 siRNA. [file 1476-4598-10-67-S3.PDF]

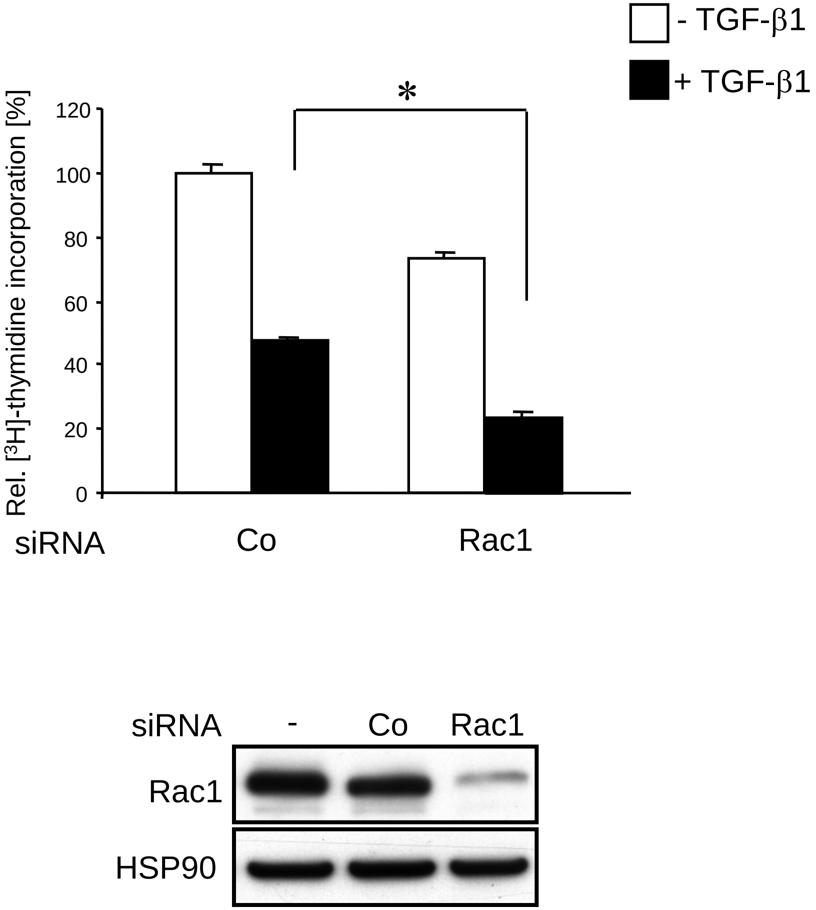

Legend to Figure S3: **SiRNA-mediated depletion of Rac1 decreases basal proliferation and enhances TGF-β1-induced growth suppression.** COLO 357 cells were transfected twice (on two consecutive days) for 24 h with 200 μM of an irrelevant control (Co), or Rac1-specific siRNA. After another 48 h in normal growth medium one half of cells was stimulated with TGF-β1 (5 ng/ml) for 24 h and subjected to [^{3}H]-thymidine incorporation assay (upper panel), while the other half was lysed and subjected to immunoblot analysis for Rac1 and HSP90 as a loading control (lower panel). The data from the graphs represent the mean ± standard deviation from a representative experiment. Three independent experiments with very similar results were performed. Asterisk, $p<0.05$.
