# Supplementary material for: Differential roles of Smad2 and Smad3 in the regulation of TGF-β1-mediated growth inhibition and cell migration in pancreatic ductal adenocarcinoma cells: control by Rac1
Source: Mol Cancer. 2011 May 30;10:67. doi: 10.1186/1476-4598-10-67 (PMC3112431; doi:10.1186/1476-4598-10-67)
Supplement: Additional file 4 — Figure S4. Reduced thymidine incorporation by Rac1 suppression is the result of reduced proliferation rather than increased apoptosis. Apoptosis assay of TGF-β1-treated PANC-1 cells transiently transfected with dn Rac1. [file 1476-4598-10-67-S4.PDF]

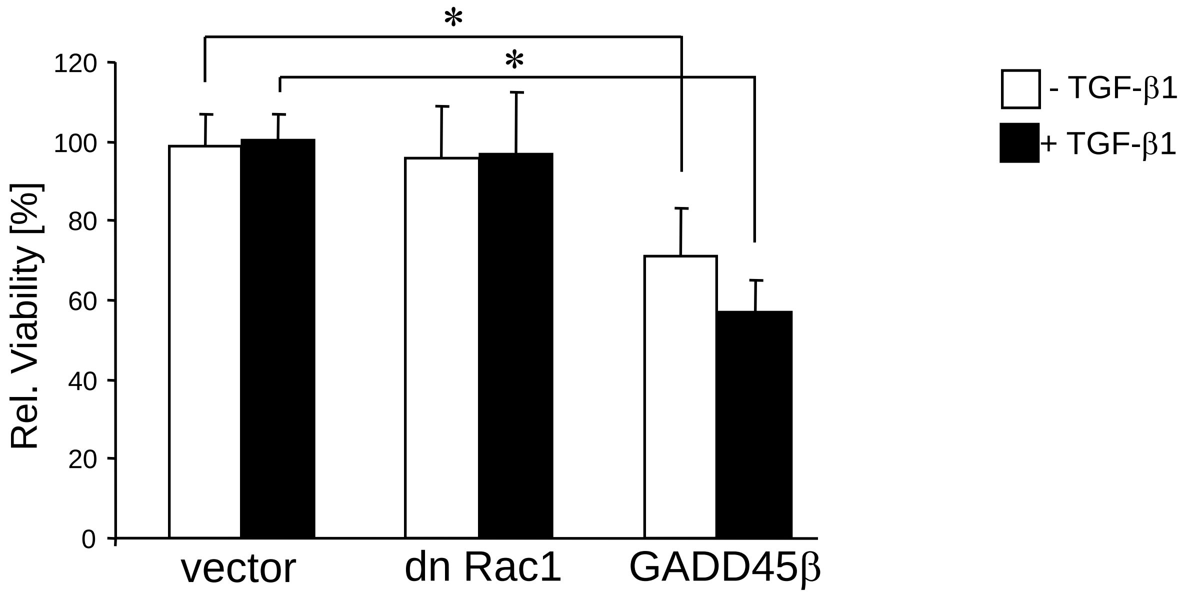

Legend to Figure S4: **Reduced thymidine incorporation by Rac1 suppression is the result of reduced proliferation rather than increased apoptosis.** Measurement of apoptosis using the JAM DNA fragmentation assay. Wild type PANC-1 cells (10,000 cells/well) were transiently transfected with expression plasmids for the indicated proteins. 24 h later cells were labeled with [^{3}H]-thymidine for 3 h and subsequently exposed to TGF-β1 for 24 h. Following harvest labeled DNA was measured and the relative viability of the various transfectants was calculated. Data are the mean ± standard deviation from six wells processed in parallel. One out of two experiments is shown. ✷, p<0.05.
