# Supplementary material for: Differential roles of Smad2 and Smad3 in the regulation of TGF-β1-mediated growth inhibition and cell migration in pancreatic ductal adenocarcinoma cells: control by Rac1
Source: Mol Cancer. 2011 May 30;10:67. doi: 10.1186/1476-4598-10-67 (PMC3112431; doi:10.1186/1476-4598-10-67)
Supplement: Additional file 5 — Figure S5. Immunoblot analysis of TGF-β1-mediated phosphorylation of (endogenous) Smad2 in COLO 357 cells in the absence or presence of the pharmacologic Rac1 inhibitor NSC23766. [file 1476-4598-10-67-S5.PDF]

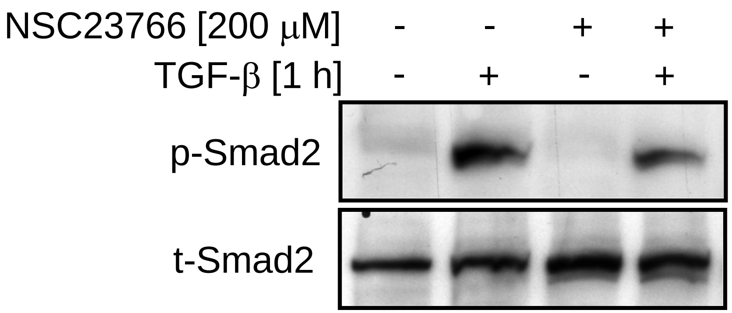

Legend to Figure S5: **TGF-β1-mediated C-terminal phosphorylation of Smad2 is sensitive to pharmacologic inhibition of Rac1.** COLO 357 cells were stimulated or not with TGF-β1 for 1 h in the absence or presence of the Rac1 inhibitor NSC23766. Crude protein extracts were fractionated by SDS-PAGE and sequentially immunoblotted for phosphorylated (p-) and total (t-) Smad2.
